# Influence of posture during mastication on body composition and nutritional intake in individuals with Down syndrome

**DOI:** 10.7717/peerj.20597

**Published:** 2026-01-15

**Authors:** Sonia Cañizares Prado, Jorge Molina-López, Maria Trinidad Moya Ruiz, Elena Planells

**Affiliations:** 1 Department of Physiology, Faculty of Pharmacy, Institute of Nutrition and Food Technology “José Matáix”, Universidad de Granada, Granada, Spain; 2 Down Syndrome Association of Granada, Granada, Spain; 3 Department of Physical Education and Sports, Faculty of Education, Psychology and Sports Sciences, Universidad de Huelva, Huelva, Spain

**Keywords:** Down syndrome, Chewing, Postural habits, Obesity, Nutritional intake, Body mass index

## Abstract

**Introduction:**

Down syndrome is associated with muscular hypotonia and feeding problems. The aim was to assess whether postural alterations during mastication had an impact on body composition, food intake and consumption.

**Methods:**

Descriptive cross-sectional study with 48 participants (8–45 years). The OMES-E protocol, anthropometric measurements of body composition and 72 h/3 days intake recording were used.

**Results:**

A total of 35.4% of participants reported being overweight or obese. Statistically significant differences were found in body mass index (BMI) (*p* = 0.022) and body fat percentage (*p* = 0.005), both being lower in those participants with postural alteration during mastication. Likewise, a significant relationship was observed between saturated fat intake and postural alteration (*p* = 0.008). Vitamin D intake was lower than the recommended levels in 77.1% of the participants and vitamin E in 95.8%. Phosphorus (P), iron (Fe) and copper (Cu) were consumed in excess by more than 50% of the sample, especially among those with postural alteration during mastication (58.3%, 45.8% and 45.8%, respectively). As for the food groups, significant differences were recorded in beef consumption, with higher intake in the group with postural alteration.

**Conclusions:**

Individuals with Down syndrome tend to present obesity and/or overweight. Those with a lower BMI and fat percentage presented postural alterations during mastication, associated with a lower overall intake than those without postural alterations. A higher intake of proteins, fats, and B-group vitamins was shown, which points to dietary behaviors that warrant closer attention due to their potential health implications.

## Introduction

Down syndrome, or trisomy 21, is a genetic disorder caused by the partial or complete presence of an extra copy of chromosome 21 (Porovic et al., 2016). It is considered the most common chromosomal disorder worldwide (De Graaf, Buckley & Skotko, 2017), with a global prevalence of 10 per 10,000 births. This condition manifests with varying degrees of intellectual disability, developmental disorders, characteristic physical features (Huete García, 2016), muscular hypotonia, cardiac complications, and diverse feeding problems (Bull & Committee on Genetics, 2011). The latter have been reported to have a higher prevalence in individuals with Down syndrome compared to their typically developing peers (Seiverling, Hendy & Williams, 2011). These feeding difficulties may stem from the anatomical-structural characteristics present in individuals with Down syndrome, such as underdeveloped mandibular growth, oral muscle hypotonia, altered and delayed dentition (González Caballero, 2014), the presence of dental malocclusions predominantly class III (Shukla et al., 2014), and temporomandibular alterations related to parafunctional habits (Salazar et al., 2016).

All these alterations impact the proper performance of the masticatory function, which is responsible for preparing food for swallowing and processing in the digestive system (Ferrari, Marinopoulou & Lydon, 2023). This process requires both lateral tongue movements and the elevation and lowering movements of the mandible, known as masticatory cycles (Le Révérend, Edelson & Loret, 2014). These specific movements facilitate the efficient mastication of the bolus, preparing it for subsequent swallowing (Shimazaki et al., 2006; Alghadir et al., 2015). There are specific postural alterations during mastication, with the most common being raised shoulder, pelvic tilt, and forward head posture (Espinosa de Santillana, 2018). On the other hand, previous studies document anterior cervical positioning as a postural alteration that leads to hyperextension of the head on the neck with mandibular retrusion (Arellano, 2002), causing poor mandibular positioning and function, constantly increasing the tension of the masticatory musculature and, consequently, producing temporomandibular dysfunction (Farias, Restani-Alves & Gandelman, 2001).

Previous research in the nondisabled population has linked obesity and overweight to poorer orofacial skills and changes in masticatory behavior (Pedroni-Pereira et al., 2016; Santos et al., 2023; Calcaterra et al., 2024). In the case of individuals with Down syndrome, although a high prevalence of overweight and obesity has been reported (Olivetti Artioli et al., 2017), the possible association with posture during masticatory function has been little explored and has not yet been conclusively established. Although, in individuals without Down syndrome, an inverse correlation of intermediate magnitude at 3 months and of small magnitude at 6 months was observed between dynamic postural balance and masticatory efficiency, suggesting the existence of a neuromuscular link between body stability and chewing function (Pai, Dhanya Kumar & Nandeeshwar, 2023). It is worth noting that individuals with Down syndrome exhibits lower chewing frequency rates, often ingesting difficult-to-masticate foods without forming an adequate food bolus (Hennequin et al., 2005). This alteration in intake could be due to hyperselectivity of food textures and/or consistencies, leading to more severe problems such as the rejection of specific foods due to poor intraoral management (Field, Garland & Williams, 2003). This is associated with a deficiency of the nutrients present in these foods, and a preference for softer foods such as carbohydrate-rich items, which could contribute to obesity (Hennequin et al., 2005). Moreover, individuals with Down syndrome in Jordan frequently exhibit insufficient intake of essential micronutrients such as zinc (Zn), selenium (Se), calcium (Ca), folates, and vitamins A and D, which can exacerbate immune, thyroid, and bone-related issues (Ali Ghazza, Al Soub & Tawfiq Ama, 2022). Similarly, Saghazadeh et al. (2017) found low levels of zinc, selenium, and calcium in individuals with Down syndrome, which were associated with increased risk of infections, thyroid dysfunction, and osteoporosis.

The study explored the possible relationship between postural alterations during chewing and body composition in individuals with Down syndrome. Unlike previous research focused exclusively on eating habits or anthropometric characteristics, this study examines outcomes related to nutritional quality and food intake in adults with Down syndrome, linking them with chewing posture. Furthermore, it provides a better understanding of the factors involved in overweight and/or obesity and imbalances in nutrient intake in these individuals.

The aims of the current study was to: (i) analyse whether the postural alterations during the masticatory function influences the body composition of individuals with Down syndrome; and (ii) analyse whether the presence or absence of postural alterations during the masticatory function was related to the adequacy of food intake and the consumption of specific food groups. In addition, the prevalence of overweight and obesity in the study sample was described to contextualize the findings. It was hypothesized that individuals with Down syndrome would exhibit a higher prevalence of overweight and/or obesity, as well as greater inadequacy in food intake and the consumption of specific food groups, promoted by the postural alteration of the masticatory function. Findings from this study could improve understanding of the relationship between posture during masticatory function and altered body mass index (BMI) with Down syndrome.

## Materials and Methods

### Participants and study design

**Figure 1:**
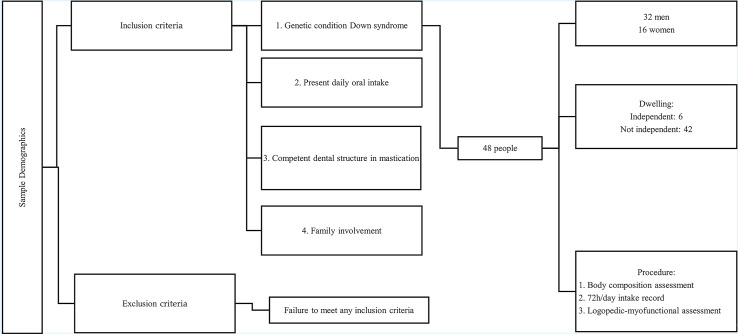
Flowchart.

A cross-sectional study with non-probabilistic convenience sampling was conducted between January 2021 and June 2023 at the Down Syndrome Association of Granada (Granada, Spain). The study included 48 individuals with Down syndrome, aged between 8 and 45 years, with a mean age and standard deviation of 22.9 (10.9) years. The sample comprised 32 males (66.7%) and 16 females (33.0%). All participants and/or their families were informed about the research objectives and they provided written informed consent. Participants were assessed in two sessions: one for measuring body composition and orofacial myofunctional function, and another for dietary intake recording. The inclusion criteria for participation were: (i) being diagnosed with Down syndrome; (ii) presenting daily oral intake; (iii) possessing a minimal dental structure for mastication; and (iv) active participation of the family during data collection. Participants who did not meet these criteria were excluded from the study. The study design, the inclusion and exclusion criteria, and the evaluation instruments used during the research are summarized in Fig. 1. The study adhered to the ethical standards established in the Declaration of Helsinki and complied with Organic Law 15/1999, of 13 December, on the Protection of Personal Data. It was approved by the Ethics Committee of the University of Granada (862/CEIH/2019).

### Instruments and procedure

#### Body composition and anthropometric measurements

Anthropometric and body composition assessments were conducted by expert personnel at the centre. Participants’ height was measured with a stadiometer in an upright posture, followed by weighing in an anatomical position with feet and hands on electrodes. Body composition was measured using the Tanita MC-980 MA Multifrequency Segmental Body Composition Analyzer (Barcelona, Spain), complying with European Standards (93/42EEC, 90/384EEC) and with NAWI Class III standards for non-automatic weighing instruments. Prior to measurements, the device was calibrated following the manufacturer recommendations (75.0 to 1,500.0 Ω). This device employs eight contact electrodes and six frequencies (1, 5, 50, 250, 500 and 1,000 kHz) to estimate the resistance and reactance across different body segments. Data included weight, fat mass, fat-free mass, muscle mass, body water, bone mineral content, sarcopenia index, phase angle, muscle quality, visceral fat, metabolic age, basal metabolic rate, proteins, and intra/extracellular water. Participants were instructed to refrain from eating or drinking 1–2 h before the test and to urinate prior to measurement.

#### 72-h food intake record

Firstly, a 72-h intake questionnaire was administered, where interviews with each participant’s family recorded food and drink consumption over 3 days using an illustrated manual with quantitative and qualitative information on food and preparation. To this end, each participant’s family recorded intake over 2 weekdays and 1 weekend day, avoiding bias from special weekend consumption habits. This 3-day dietary diary is a reference method for assessing intake (Barrett-Connor, 1991). The use of this log has been based on studies indicating that longer logging periods, such as 72 h, produce data comparable to weighed food assessments, thereby improving the validity of dietary intake estimates (Jago et al., 2019). This research was also based on the literature that highlights the use of daily records as a source for measuring dietary variability between weekdays and special events (weekends) (Baranowski, 2012). Likewise, previous studies corroborate the use of this instrument in their methodology (Molina-López et al., 2021; Roccatello et al., 2021; Skrzypek et al., 2021; Herrera-Quintana et al., 2022; Vázquez-Lorente et al., 2022; Molina-López et al., 2024).

Data on food and beverage consumption were converted into absolute values referring to energy intake, macro and micronutrient intake, and the percentage of adequate intake for each nutrient (recommended daily allowance) using Dietowin software (version 11.0; Barcelona, Spain). The nutritional data obtained were compared with the Dietary Reference Intakes (DRIs) for the Spanish population (Report of the Scientific Committee of the Spanish Agency for Food Safety and Nutrition, AESAN, 2019) (Martinez-Hernández et al., 2020). The adequacy or inadequacy of intake was determined by comparing the actual intake of each participant with the recommended intake for each nutrient, with two cut-off points: insufficient intake (less than 66.6% of DRI) and excessive intake (above the DRI). Additionally, the specific degree of inadequate intake in the participants was calculated by determining the total number of macronutrients and micronutrients with insufficient intake, and three cut-off points were established: slightly inadequate intake (inadequate intake of one to five nutrients), moderately inadequate intake (insufficient intake of six to ten nutrients), and highly inadequate intake (insufficient intake of more than ten nutrients) (Marí-Bauset et al., 2015). The three cut-off points described above were also considered for those macronutrients and micronutrients that were over-consumed.

#### Myofunctional assessment

The OMES-E test was used for functional oral-motor assessment. The validated protocol was used to reduce measurement bias. The OMES-E test has demonstrated high reliability, with intraclass reliability coefficients between 0.86 and 0.93, as well as good agreement between evaluators (r between 0.70 and 0.84). In addition, it has demonstrated consistency in both paediatric and adult populations, supporting its clinical applicability (Felício et al., 2010). The OMES-E protocol consists of an orofacial myofunctional assessment with expanded scores. This protocol evaluates the appearance, posture, and mobility of the face, cheeks, jaw, tongue, and lips, as well as the functions of breathing, chewing, and swallowing in terms of performance and duration. Global scores range from 1 to 4, with 4 = normal, 3 = mild dysfunction, 2 = moderate dysfunction, and 1 = severe dysfunction. To specifically assess oral functions such as mastication and swallowing, ordinal scores are used to differentiate the presence or absence of functional alterations. In particular, regarding mastication, a score of 2 indicates an adequate pattern and coordination without signs of compensation or difficulty. In contrast, a score of 1 indicates the presence of alterations, such as food escape, inappropriate body posture, or compensatory movements of other body parts. This assessment of body posture during chewing was executed following the criteria established in the protocol, which includes direct clinical observation of the position of the head, neck, shoulder girdle, and trunk. According to OMES-E, posture alterations are classified dichotomously as “present” or “absent,” considering the presence of postural alterations when visible deviations are identified, such as head tilt or rotation, shoulder asymmetry, or trunk misalignment. The absence of alterations corresponds to a symmetrical and aligned posture at rest. This qualitative assessment, based on standardized protocol criteria, ensures reliable and reproducible application for orofacial functional assessment in the context of mastication.

For the assessment, each participant sat in a chair with a backrest facing the assessor and in front of a mirror that allowed proprioception of movements. The protocol was then explained to each participant. Initially, the assessment was based on the observation of the anatomical structures and, subsequently, muscle mobility was assessed in specific oral movements. To do this, the examiner provided a model for the participants to imitate, and a mirror was also used to help the participants understand the specific movement they had to reproduce.

A cookie was used as a standardized solid food, adapted to the dietary needs of each participant (sugar-free, gluten-free, egg-free and dairy-free). Liquid intake was assessed with a glass of water to observe intraoral manipulation. Both solid and liquid intake was recorded with a 12-megapixel camera placed 1 m away from the participant. Prior to initiating intake, participants were positioned with their backs supported on the chair and feet on the floor to promote postural balance. The principal investigator recorded any postural movements during chewing and/or swallowing by analyzing the recorded videos. Intake time was noted from when the food was introduced into the oral cavity until the last swallow was recorded.

### Data analysis

The data were analyzed with the SPSS version 28.0 statistical package (SPSS, Inc., Chicago, Illinois, USA). For sample characteristics, descriptive statistics were performed using the mean, standard deviation, minimum and maximum, as well as percentages of the study data. These data were calculated for the total sample, as well as for the men and women who participated in the study. All analyses were performed taking into account the variable presence/absence of postural alteration during mastication. The normality of continuous variables was assessed using the Shapiro-Wilk test. In addition, histograms were inspected to visually examine the shape of the distributions. Since several variables did not follow a normal distribution, the dataset was considered nonparametric. For this purpose, a nonparametric test for independent samples was performed through the median and quartiles 25–75 for both the total sample and for the groups established between presence/absence of postural alteration during mastication. Body composition, nutritional intake and food group consumption were analyzed using the Mann-Whitney U test and the Kolmogorov-Smirnov Z test. At the level of nutritional intake, energy, macronutrient, vitamin and mineral inadequacy and adequacy were also calculated, and the chi-square test was used to calculate differences between groups. Finally, total macronutrient and micronutrient deficiency and excess were calculated and groups with and without postural alterations during mastication were compared. Statistical level of significance was set at *p* < 0.05.

## Results

### Sample characteristics

Table 1 shows the participant characteristics. The body composition analysis revealed comparable mean values between men and women, with an overall BMI indicating a normal weight range (Table 1). According to the values established by the WHO, BMI was classified as underweight (<18.5), normal weight (18.5–24.9), overweight (25.0–29.9) and obese (≥30). After analysis, 10.4% of the total participants were classified as underweight (10.4% of men and 0% of women), 54.2% as healthy weight (35.4% of men and 18.8% of women), 25.0% as overweight (12.5% of men and 12.5% of women), and 10.4% as obese (8.3% of men and 2.1% of women). The mean percentage of body fat was 21.0 ± 8.09% (19.0 ± 6.9 in men and 24.9 ± 9.1 in women). Regarding the living habits of the participants, only 12.5% lived independently, 8.3% being men and 4.2% women.

**Table 1:** Sample characteristics.

	Men (*n* = 32)				Women (*n* = 16)				Total (*n* = 48)			
	Mean	SD	Min	Max	Mean	SD	Min	Max	Mean	SD	Min	Max
Age (years)	24.9	11.8	8	45	26.2	11.4	10	45	22.9	10.9	8	45
Weight (kg)	54.4	14.8	25.7	86.4	54.2	10.4	37.7	78.6	54.3	13.4	25.7	86.4
Height (cm)	149.8	11.2	122	166	146.1	7.9	135	160	148.5	10.3	122	166
Basal metabolism (kcal)	1,486.6	173.6	1,120	1,805	1,298.2	132.5	1,059	1,564	1,423.8	183.1	1,059	1,805
Fat mass (%)	19.0	6.9	8.5	32.7	24.9	9.1	7.2	38.7	21.0	8.09	7.2	38.7
Lean mass (kg)	41.6	10.4	18.5	55.8	38.2	6.1	28.8	50.8	40.4	9.27	18.5	55.8
Bone mineral (kg)	2.2	0.5	1.1	2.9	2.1	0.3	1.6	2.7	2.19	0.45	1.1	2.9
BMI (kg/m^2^)	23.7	5.1	16.5	37.9	25.4	4.6	19.4	38.4	24.4	4.93	16.5	38.4
BMI (WHO)	***n***		**%**		***n***		**%**		***n***		**%**	
Underweight	5		10.4		0		0.0		5		10.4	
Healthy weight	17		35.4		9		18.8		26		54.2	
Overweight	6		12.5		6		12.5		12		25.0	
Obesity	4		8.3		1		2.1		5		10.4	
Dwelling	***n***		**%**		***n***		**%**		***n***		**%**	
Independent	4		8.3		2		4.2		6		12.5	
Non-independent	28		58.3		14		29.2		42		87.5	

**Note:**

Continuous variables were expressed as mean, standard deviation (std. dev.), minimum (min.) and maximum (max.). Categorical variables were expressed as number and percentage of subjects. Descriptive analysis was performed with frequencies. Abbreviations: kg, kilograms; cm, centimetres; kcal, kilocalories; BMI, body mass index.

### Body composition

Table 2 shows the differences of body composition based on the presence or absence of postural alterations during chewing. Results showed that 29 participants exhibited postural alterations during chewing. It was observed that subjects with postural alteration had a lower BMI (*p* = 0.022) and lower body fat percentage (*p* = 0.005) than their peers without postural alteration during mastication. No differences were observed for the other analysed variables.

**Table 2:** Relationship between body composition and postural alteration during mastication.

	Total (*n* = 48)		Presence (*n* = 29)		Absence (*n* = 19)		Z	*vP*
	Median	25th–75th	Median	25th–75th	Median	25th–75th		
Weight (kg)	54.3	44.1–64.0	49.9	42.6–62.3	56.4	47.3–66.4	–1.634	0.102
Height (cm)	151.0	142.2–156.0	151.0	142.0–156.0	147.0	142.0–158.0	–0.190	0.849
BMI (kg/m2)	23.5	21.9–26.3	22.5	20.5–24.9	25.0	22.3–29.5	–2.298	0.022
Basal metabolism (kcal)	1,426.0	1,268.2–1,585.7	1,465.0	1,279.0–1,540.0	1,292.0	1,265.0–1,604.0	–0.285	0.776
Fat mass (%)	21.0	14.4–26.9	15.6	12.9–24.4	24.8	20.7–31.8	–2.815	0.005
Lean mass (kg)	40.5	33.8–48.8	39.4	33.3–46.3	40.5	34.2–50.9	–0.611	0.541
Bone mineral (kg)	2.20	1.90–2.60	2.20	1.85–2.45	2.20	1.90–2.70	–0.486	0.627
Total body water (kg)	30.6	26.1–38.4	32.0	25.5–35.0	30.6	26.4–40.8	–0.580	0.562

**Note:**

Continuous variables are expressed as median and interquartile range (25th–75th percentile). A nonparametric test for independent samples was performed using the Mann-Whitney U test (considered significant if *p* < 0.05). **Z** = Mann-Whitney U test statistic. **vP** = Statistical significance was set at *p* < 0.05. Abbreviations: kg, kilograms; cm, centimetres; kg/m^2^, kilograms per square meter; kcal, kilocalories.

### Nutritional intake

Table 3 compares energy, macro, and micronutrient intake and intake adequacy in individuals with and without postural alteration during mastication. A significant association was observed between saturated fat intake and postural alteration (*p* = 0.008, chi-square test). Specifically, 22.9% of participants without postural alteration showed excessive intake, compared to 12.5% among those with postural alteration. In contrast, no significant difference was found in the median saturated fat intake in grams between groups. No significant differences were found for other macronutrients, but 81.3% of participants had excess protein intake. Additionally, 25% of participants had insufficient energy intake than recommended for their age and sex.

**Table 3:** Comparative analysis between postural alteration during mastication and adequacy of micro and macronutrient intake.

	Total (*n* = 48)				Presence (*n* = 29)				Absence (*n* = 19)				$\chi^{2}$	Z	*vP*
	Median	25th–75th	Inadequacy	Excess	Median	25th–75th	Inadequacy	Excess	Median	25th–75th	Inadequacy	Excess			
**Macronutrients**															
Energy (Kcal)	1,896	1,563.8–2,195.0	12 (25.0)	10 (20.8)	1,890	1,490.5–2,191.5	9 (18.8)	6 (12.5)	1,970	1,619.0–1,970.0	3 (6.3)	4 (8.3)	0.464	–0.548	0.584
Proteins (g)	84.2	71.3–99.2	1 (2.1)	39 (81.3)	85.5	76.6–105.3	0 (0)	25 (52.1)	79.7	58.0–98.1	1 (2.1)	14 (29.2)	0.348	–1.444	0.149
Carbohydrates (g)	239.8	212.4–296.9	7 (14.6)	1 (2.1)	239.6	213.2–300.1	4 (8.3)	1 (2.1)	241.5	210.3–297.9	3 (6.3)	0 (0)	0.708	–0.053	0.958
Lipids (g)	64.4	45.3–80.8	4 (8.3)	8 (16.7)	56.8	42.0–74.3	4 (8.3)	3 (6.3)	78.0	52.1–82.5	0 (0)	5 (10.4)	0.112	–1.465	0.143
Sugar (g)	72.9	53.9–104.9	–	11 (22.9)	67.5	53.0–105.1	–	7 (14.6)	74.1	58.1–105.6	–	4 (8.3)	0.804	–0.527	0.598
Fiber (g)	17.9	14.3–22.4	13 (27.1)	16 (33.3)	18.2	15.1–22.6	7 (14.6)	9 (18.8)	16.6	14.2–22.6	6 (12.5)	7 (14.6)	0.651	–0.506	0.613
Saturated fats (g)	18.6	13.1–27.8	–	17 (35.4)	17.0	11.6–24.2	–	6 (12.5)	22.3	14.9–33.6	–	11 (22.9)	0.008	–1.887	0.059
Cholesterol (g)	0.3	0.2–0.4	–	0 (0)	0.26	0.2–0.4	–	0 (0)	0.3	0.2–0.4	–	0 (0)	–	–0.084	0.933
**Vitamins**															
Vitamin A (µg)	516.5	363.0–823.2	19 (39.6)	15 (31.3)	524	358.5–822.5	12 (25.0)	8 (16.7)	509	366.0–834.0	7 (14.6)	7 (14.6)	0.794	–0.105	0.916
Vitamin B_1_ (mg)	1.8	1.5–2.1	1 (2.1)	46 (95.8)	1.77	1.5–2.2	1 (2.1)	27 (56.3)	1.9	1.4–2.2	0 (0)	19 (39.6)	0.505	–0.432	0.666
Vitamin B_2_ (mg)	1.8	1.4–2.2	2 (4.2)	41 (85.4)	1.84	1.4–2.3	1 (2.1)	24 (50.0)	1.8	1.3–2.0	1 (2.1)	17 (35.4)	0.621	–0.464	0.643
Vitamin B_3_ (mg)	14.8	12.1–20.3	4 (8.3)	23 (47.9)	14.8	12.2–21.5	3 (6.3)	14 (29.2)	14.5	12.0–20.0	1 (2.1)	9 (18.8)	0.798	–0.232	0.817
Vitamin B_6_ (mg)	1.7	1.3–2.2	0 (0)	34 (70.8)	1.7	1.3–2.3	0 (0)	20 (41.7)	1.5	1.3–2.1	0 (0)	14 (29.2)	0.725	–0.801	0.423
Vitamin B_12_ (µg)	4.2	3.0–5.4	2 (4.2)	41 (85.4)	4.08	3.0–5.4	1 (2.1)	25 (52.1)	4.7	2.9–5.4	1 (2.1)	16 (33.3)	0.953	–0.19	0.850
Vitamin C (mg)	126	82.1–169.3	2 (4.2)	40 (80.3)	135.1	100.3–190.4	2 (4.2)	24 (50.0)	110.5	77.3–164.6	0 (0)	16 (33.3)	0.453	–1.297	0.195
Vitamin D (µg)	2	1.0–8.0	37 (77.1)	10 (20.8)	2	1.0–30.5	20 (41.7)	8 (16.7)	1	1.0–5.0	17 (35.4)	2 (4.2)	0.236	–1.182	0.237
Vitamin E (mg)	0.2	0.1–0.3	46 (95.8)	2 (4.2)	0.2	0.1–0.4	28 (58.3)	1 (2.1)	0.1	0.0–0.2	18 (37.5)	1 (2.1)	0.758	–1.804	0.071
Vitamin K (µg)	60.8	36.8–126.5	11 (22.9)	24 (50.0)	57.9	40.8–120.6	5 (10.4)	14 (29.2)	61.8	35.2–200.0	6 (12.5)	10 (20.8)	0.279	–0.2	0.841
**Minerals**															
Sodium (mg)	2,404	1,650.0–2,165.0	2 (4.2)	39 (81.3)	2,111	1,582.5–3,197.5	1 (2.1)	23 (47.9)	2,524	1,828.0–3,147.0	1 (2.1)	16 (33.3)	0.787	–0.601	0.548
Potassium (mg)	2,652	2,393.5–3,079.2	7 (14.6)	0 (0)	2,802	2,475.5–3,125.0	3 (6.3)	0 (0.00)	2,506	2,322.0–3,010.0	4 (8.3)	0 (0)	0.304	–1.307	0.191
Calcium (mg)	845.5	702.7–996.0	13 (27.1)	13 (27.1)	850	694.5–1,003.5	9 (18.8)	9 (18.8)	789	736.0–950.0	4 (8.3)	4 (8.3)	0.398	–0.633	0.527
Magnesium (mg)	265	231.2–316.0	5 (10.4)	14 (29.2)	282	240.0–314.0	2 (4.2)	6 (12.5)	257	228.0–331.0	3 (6.3)	8 (16.7)	0.110	–0.39	0.696
Phosphorus (mg)	1,272	1,080.7–1,439.7	0 (0)	46 (95.8)	1,288	1,127.0–1,481.5	0 (0)	28 (58.3)	1,255	1,010.0–1,430.0	0 (0)	18 (37.5)	0.758	–0.706	0.480
Iron (mg)	14.5	12.3–20.3	7 (14.6)	35 (72.9)	14.4	12.9–21.1	2 (4.2)	22 (45.8)	15.22	10.6–18.8	5 (10.4)	13 (27.1)	0.112	–0.559	0.576
Copper (mg)	1.4	1.2–1.8	1 (2.1)	35 (72.9)	1.4	1.2–1.7	1 (2.1)	22 (45.8)	1.32	1.1–1.8	0 (0)	13 (27.1)	0.526	–0.2	0.841
Zinc (mg)	8.8	7.5–10.2	5 (10.4)	18 (37.5)	8.8	7.3–10.4	3 (6.3)	11 (22.9)	8.8	8.0–10.0	2 (4.2)	7 (14.6)	0.997	–0.2	0.841
Manganese (mg)	3.3	2.4–5.0	4 (8.3)	30 (62.5)	3.5	2.4–5.0	3 (6.3)	21 (43.8)	3.0	2.5–5.5	1 (2.1)	9 (18.8)	0.079	–0.042	0.966

**Note:**

Continuous variables were expressed as median and quartiles 25th–75th. Categorical variables were expressed as number and percentage of subjects. Comparison between groups (with and without postural disturbance during chewing) was performed using the Chi–square test for inadequacy or excess of each nutrient. Bonferroni–corrected significance levels were calculated for multiple comparisons of nutritional analyses of macronutrients, vitamins and minerals (considered significant if *p* < 0.05). The Nutritional Intake (INR) percentages were calculated according to the Report of the Scientific Committee of the Spanish Agency for Food Safety and Nutrition (AESAN) for the Spanish population (Martinez-Hernández et al., 2020). Inadequate intake was determined by comparing the actual intake of each participant with the recommended intake for each nutrient, with two cut–off points: an intake below 66.6% of the INR was classified as “insufficient intake”; an intake above 100% of the INR was classified as “excessive intake”. Abbreviations: Kcal, kilocalories; g, grams; mg, milligrams; µg, micrograms.

Regarding vitamin intake, although no significant differences were found between groups, over 50% of the sample exhibited excessive intake of vitamins. Values higher than those recommended were observed for vitamin B_1_ (95.8% of the total, 56.3% for the group with postural alteration), vitamin B_2_ (85.4% of the total, 50% for individuals with postural alteration), vitamin B_6_ (70.8% of the total, 41.7% for the group with postural alteration), vitamin B_12_ (85.4% of the total, 52.1% for the group with postural alteration), vitamin C (80.3% of the total, 50.0% for the group with postural alteration), and vitamin K (50.0% of the total, 29.2% for the group with postural alteration). Conversely, vitamin D and vitamin E were consumed below recommended levels in over 50% of the sample (77.1% of the total, 41.7% for the group with postural alteration; and 95.8% of the total, 58.3% for the group with postural alteration, respectively).

Finally, while no significant differences were found in mineral intake between groups, there was a notable trend (*p* = 0.079) towards excessive manganese (Mn) intake, with 43.8% of those with postural alteration during mastication exceeding recommended levels compared to 18.8% without postural alteration. P, Fe, and Cu were consumed in excess by over 50% of the sample (95.8%, 72.9%, and 72.9% respectively), particularly prevalent among those with postural alteration (58.3%, 45.8%, and 45.8% respectively). No mineral intake fell below recommended levels in over 50% of the sample, yet Ca had the highest percentage (27.1%) with insufficient intake.

### Food groups

Table 4 presents the comparative analysis of daily food intake by food groups based on the presence/absence of postural alteration during mastication. Differences were found in beef/veal intake (*p* = 0.005), with a higher intake in the group with postural alteration, with a difference of 42.0 g per day. Rice and semolina intake showed a trend towards significance (*p* = 0.055), being higher in the non-alteration group. Except for pasta, white meat, and white fish intake, individuals with postural alteration had lower median food intake. Additionally, participants reported little to no daily intake of certain food groups, including cereals, tubers, pork, lamb, oily fish, nuts, and fatty cheeses during the assessment period (72 h/3 days).

**Table 4:** Comparative analysis between postural alteration during mastication and daily nutrient quantification by food groups.

Food groups/day	Total (*n* = 48)		Presence (*n* = 29)		Absence (*n* = 19)		*vP*
	Median	25th–75th	Median	25th–75th	Median	25th–75th	
Packaged juices (ml)	0.0	0.0–103.8	0.0	0.0–156.7	0.0	0.0–66.7	0.370
Eggs and egg derivates (g)	21.7	0.3–57.0	21.0	3.5–56.3	40.0	0.0–58.7	0.633
Sausages (g)	25.0	6.3–49.8	25.0	5.8–48.3	25.0	6.3–51.7	0.808
Vegetables and greens (g)	196.3	136.7–257.3	192.0	154.5–263.8	200.7	132.7–247.3	0.238
Legumes (g)	25.5	0.0–66.3	23.3	0.0–51.0	28.7	0.0–73.3	0.706
Fresh fruit (g)	171.7	81.7–285.8	152.0	53.3–293.3	193.3	132.0–273.3	0.187
Rice and semolina (g)	26.5	0.0–42.2	20.0	0.0–33.3	33.3	24.3–48.7	0.055
Pasta (g)	47.3	0.0–113.7	65.0	0.0–104.3	0.0	0.0–160.0	0.776
Whole milk products (ml)	213.7	83.3–293.4	125.0	77.7–341.0	250.7	91.3–291.7	0.598
Non–fat dairy products (ml)	83.3	5.8–258.6	83.3	4.3–254.7	83.3	8.3–273.7	0.775
Bread and complements (g)	104.0	74.1–149.6	101.7	71.7–169.0	105.7	78.3–143.3	0.924
Beef/Calf (g)	0.0	0.0–31.6	8.0	0.0–42.0	0.0	0.0–0.0	0.005
White meats (g)	40.2	12.0–101.8	60.0	12.0–117.3	24.3	12.0–53.7	0.170
White fish (g)	18.0	0.0–67.2	32.3	0.0–87.5	0.0	0.0–42.0	0.076
Crustaceans/shellfish/molluscs (g)	0.0	0.0–8.7	0.0	0.0–3.0	0.0	0.0–22.0	0.043
Sugars (g)	11.0	0.0–33.9	10.3	0.0–36.8	12.3	3.3–23.7	0.890
Chocolate and derivatives (g)	0.0	0.0–34.3	0.0	0.0–19.8	2.0	0.0–69.3	0.236
Spices and flavourings (g)	1.3	0.0–4.0	0.3	0.0–3.8	2.0	0.0–4.7	0.646
Flours (g)	0.0	0.0–5.3	0.0	0.0–3.0	1.3	0.0–8.0	0.210
Oils and fats (g)	15.8	8.9–29.8	13.7	9.2–21.3	21.3	8.7–45.0	0.191

**Note:**

Continuous variables were expressed as median and quartiles 25th–75th. Categorical variables were expressed as number and percentage of subjects. Daily intake of each food group was calculated and a nonparametric test for independent samples was performed using the Mann–Whitney U test (considered significant if *p* < 0.05). Those food groups in which daily intake was 0 were eliminated. ml, millilitres; mg, milligrams.

### Nutritional insufficiency and excess

Table 5 shows the results concerning inadequacy and nutritional excess of macronutrients and micronutrients in relation to postural alteration during mastication. Regarding inadequacy, no differences were found between groups (macronutrients *p* = 0.592; vitamins *p* = 0.604; minerals *p* = 0.705), but higher values of inadequacy were observed in the average intake of vitamins (Mdn = 2.58), being superior in the group of individuals without alteration during mastication (Mdn = 2.68 *vs*. 2.52).

**Table 5:** Nutritional inadequacy and excess and postural disturbance during mastication in people with Down syndrome.

Nutritional inadequacy	Total (*n* = 48)		Presence (*n* = 29)		Absence (*n* = 19)			Z	*vP*
	Mean	SD	Mean	SD	Mean	SD	x^2^		
Macronutrients	0.8	0.9	0.8	0.9	0.7	0.8	0.498	−0.535	0.592
Vitamins	2.6	1.2	2.5	1.4	2.7	1.0	0.148	−0.518	0.604
Minerals	0.9	1.6	0.8	1.6	1.1	1.6	0.497	−0.379	0.705
**Excess intake**									
Macronutrients	1.9	0.9	1.6	0.7	2.2	1.1	0.094	−1.396	0.163
Vitamins	5.8	1.7	5.4	1.6	5.8	1.6	0.723	−0.396	0.692
Minerals	4.8	2.0	4.3	1.4	4.5	2.2	0.212	−0.523	0.601

**Note:**

Continuous variables were expressed as mean and standard deviation. The sum of all those macro and micronutrients where the participants presented inadequacy and excess were summed and a comparison between groups (with and without postural alteration during mastication) was made using the Chi-square test. Bonferroni-corrected significance levels were calculated (significant if *p* < 0.05). Finally, a nonparametric test for independent samples was performed using the Mann-Whitney U test.

In relation to excess, no differences were found between groups (macronutrients *p* = 0.163; vitamins *p* = 0.692; minerals *p* = 0.601), but higher values of excess were found in vitamin intake (Mdn = 5.75), being higher in the group of individuals without alteration during mastication (Mdn = 5.84 *vs*. 5.32).

## Discussion

The aim of this study was to (i) analyse whether postural alterations during the masticatory function influences body composition in individuals with Down syndrome, and (ii) assess whether these alterations are related to dietary adequacy and the consumption of specific food groups. The main findings of the study demonstrated that 25% of participants were overweight and 10.4% were obese. Although this combined prevalence of 35% falls at the lower end of the range reported in previous studies (23–70%) (Bertapelli et al., 2016), and is lower than the 66% reported by others studies (Real de Asua et al., 2014), these differences may be explained by variations in sample characteristics and assessment methods. On the other hand, excess saturated fat intake was more frequent in participants without postural alterations (22.9%), and 81.3% of participants had excess protein intake. No significant differences in nutrient inadequacy were found, although inadequacy of vitamins D and E ranged from 41.7% to 58.3% in participants with postural alterations. Significant trends were also observed in the intake of beef/veal and rice and semolina.

Previous research on a sample of 42 adolescents without intellectual disabilities studied the potential relationship between obesity/overweight (*via* BMI) and masticatory behaviours, demonstrating an inverse relationship between obesity/overweight and mastication time, understood as masticatory cycles per second, with individuals having higher BMI exhibiting shorter mastication times (Idris et al., 2021). Other authors have also shown that head and neck posture influence masticatory muscle activity (Gadotti et al., 2020). However, no literature directly examines the relationship between masticatory function and postural alterations during mastication that could influence BMI. Our study found significant differences in BMI and fat percentage between groups, showing lower BMI and fat percentage in those individuals with postural alteration. Likewise, our results suggest that postural alterations during mastication could lead to a slower chewing pattern, associated with a lower BMI. In this sense, previous research highlights that when the chewing time is longer, there is a tendency to eat less because the food processing information is transmitted to the nervous system and hormones that generate the sensation of satiety are secreted. Therefore, they suggest that those who chew faster may tend to eat more food (Scudine et al., 2016; Pedroni-Pereira et al., 2016).

In relation to energy intake, participants with Down syndrome in a Polish study reported adequate energy intake in the majority of the sample, while 27.8% of participants exceeded the estimated energy needs for their weight, age and gender (Skrzypek et al., 2021). Our study shows similar results for excessive intake, with 20.8% of our sample population exceeding the recommended amount. In contrast, the Polish study reported that the rest of the study population met the reference intake, whereas our data found that 25% of people with Down syndrome did not meet the recommended energy standards and thus had energy inadequacy. In relation to the intake of macro and micronutrients, available studies in the population with intellectual disabilities have shown disparaged results (Skrzypek et al., 2021). For example, a Brazilian study compared children and adolescents with Down syndrome with gender- and age-matched controls, reported excessive intake of carbohydrates (78.9%), protein (94.7%) and fat (84.2%) (Magenis et al., 2018). Other studies have also reported significantly higher amounts for the three main macronutrients in adolescents with Down syndrome (AbdAllah et al., 2013). Another Greek study involved 18 girls and 16 boys with Down syndrome, aged between 2 and 18 revealed a high protein intake (Grammatikopoulou et al., 2008). On the other hand, a Spanish study reported data with energy intakes of 45.6% and 43.3% from carbohydrates, 16.3% and 18.8% from protein, and 38.1% and 37.9% from fat in men and women with Down syndrome, respectively (Soler Marín & Xandri Graupera, 2011). In this sense, our data show that 81.3% of the sample had an excessive protein intake, in agreement with previous evidence. It should be noted that no previous studies have analysed the relationship between macro- and micronutrient intake in relation to postural character during mastication. In this regard, our study shows significant differences in the intake of saturated fats, which were ingested in excess by 10.4% more of the sample belonging to the group without postural alteration during mastication.

Likewise, there is higher variability in vitamin intake in populations with intellectual disabilities. Our data revealed an intake higher than the recommendations in more than half of the sample for vitamins B_1_, B_2_, B_6_ and B_12_, these results that corroborate to those obtained in previous studies (Skrzypek et al., 2021). In this regard, existing evidence suggests that excessive intake may play an important role in the development of obesity (Zhou & Zhou, 2014). In contrast, another Polish study reported insufficient intakes of group B in the Down syndrome population (Mazurek & Wyka, 2015). In terms of vitamin C intake, previous research in individuals with Down syndrome has reported excess intake in 96.6% of participants (AbdAllah et al., 2013), the results are close to those obtained in our study (80.3%). Regarding the adverse effects related to the intake of acute doses of this vitamin, previous studies have highlighted gastrointestinal symptoms as the most common (National Health and Medical Research Council & New Zealand Ministry of Health, 2006). Finally, our results provided information on insufficient intake of vitamins E and D, with vitamin E being the most inadequate (95.8% of participants), in line with previous research (Grammatikopoulou et al., 2008). This vitamin E deficiency is directly related to a poorer immune response to infectious diseases (Lee & Han, 2018). Similarly, a high proportion of the participants in our study (77.1%) reported low vitamin D intake, which is consistent with the results of previous research (Grammatikopoulou et al., 2008; Millares-Pérez et al., 2016; Magenis et al., 2018) with consequent long-term health problems (Galesanu & Mocanu, 2015). Specifically, vitamin D deficiency has been related to osteoporosis, muscle weakness and decreased immune function (Olivo-Torres et al., 2023).

Regarding mineral intake, studies report a high percentage of inadequacy in relation to the intake of minerals such as Ca (Grammatikopoulou et al., 2008; AbdAllah et al., 2013; Skrzypek et al., 2021), similar to our study, where 27.1% of the participants had an intake below the recommendation. People with Down syndrome may be at higher risk of osteoporosis than the general population, who would recommend calcium supplementation if Ca levels are low in order to improve bone health in this group (García, Valero & Riancho, 2016). Also, other studies have reported inadequate Fe intake (Bertoli et al., 2006), contrary to what was observed in our participants, where 72.9% of the subjects had a high Fe intake. Finally, 95.8% of the participants in our study reported a high phosphorus intake, which is similar to previous studies (Skrzypek et al., 2021). It is noteworthy that there is a lack of literature analysing the relationship between postural alteration during mastication and macro- and micronutrient intake, so although no significant differences were found when analysing this intake, we suggest the need to investigate this relationship.

In terms of intake by food group, our results found a trend towards statistical significance for certain foods such as rice and semolina when comparing between groups, with a higher intake of this food group for the subjects who did not present disturbance during chewing. Previous studies have classified rice as a “grainy/sandy” food (Ross, Bernhard & Smith-Simpson, 2019) that could hinder bolus formation in the oral cavity, and therefore be substituted by foods of a consistency and/or texture that do not pose a challenge for chewing. On the other hand, our results also show a lower mean intake of most of the food groups studied in individuals with postural alteration during mastication, which could be related to the lower BMI values found in this group. Finally, it is worth noting the low intake of blue fish, cereals, and nuts, as none of these healthy groups were consumed during the three study days. This finding, together with the generally unbalanced dietary pattern often observed in individuals with Down syndrome—characterised by low consumption of fruits, vegetables, whole grains—highlights the need to align their diet with general nutritional recommendations (Gruszka & Włodarek, 2024). In this regard, the data obtained differ from the guidelines proposed by organizations such as the Spanish Agency for Food Safety and Nutrition (AESAN) (Agencia Española de Seguridad Alimentaria y Nutrición, 2022), which recommend consuming oily fish three times a week (120–150 g per portion). Likewise, it is recommended that cereals should be consumed three to six times a day (40–80 grams per portion). Finally, the amount of nuts and dried fruit that is considered healthy is around three times a week (20–30 grams per portion). This low intake of the above food groups could have negative effects on overall health. Specifically, previous studies have linked oily fish intake to a reduced risk of coronary events and a 30% decrease in mortality from cardiovascular disease (Burr et al., 1989), which, together with the higher incidence of congenital heart disease in the Down syndrome population (de Rubens Figueroa et al., 2003), may pose a greater risk to overall health. Similarly, low intakes of cereals and nuts in people with Down syndrome have also been found in previous research (Millares-Pérez et al., 2016). This low intake of cereals, a potential source of fibre, could have negative health effects, as adequate intake of cereals, preferably whole grains, is associated with a lower risk of coronary heart disease, diabetes, obesity and certain gastrointestinal disorders (Prasadi & Joye, 2020). Finally, nuts are an important source of vitamins and antioxidants and their consumption has been found to be inversely associated with the risk of various diseases such as diabetes, cardiovascular disease, hypertension, dyslipidaemia (Blomhoff et al., 2006; Grosso & Estruch, 2016), and improved cognitive function as an antioxidant protector (Arias-Fernández et al., 2019). People with Down syndrome manifest alterations in cognitive functioning throughout all stages of life, being associated in late adulthood with the onset of Alzheimer’s type dementia (Grieco et al., 2015), so it is considered important to eat foods rich in antioxidants that can provide all the aforementioned benefits to cognitive, cardiovascular, gastrointestinal function and general health.

This study has certain limitations. The small sample size limits generalization to the broader Down syndrome population, and the uneven distribution of age and sex among participants prevents analysis by these variables. Future studies should ensure a homogeneous sample. The 72h/3-day intake register relies on family participation and captures only 3 days of diet, missing seasonal variation. A longitudinal food frequency register would improve data collection. Additionally, lack of standardized nutritional recommendations across countries complicates comparisons. Finally, no studies have considered postural alterations during mastication, which should be explored in future research.

## Conclusions

The study showed that people with Down syndrome tended to be obese and/or overweight. Those with lower BMI and fat percentage presented postural alterations during mastication. Participants without postural disturbances tended to consume excessive fat. There was a generalized excess in the intake of protein and vitamins B_1_, B_2_, B_6_, B_12_, C and K, while vitamins E and D were the least consumed. In minerals, an excessive intake of P, Fe and Cu was observed, especially in those with postural alterations. The least consumed mineral was Ca. In addition, participants with postural alterations consumed less food, with minimal or no intake of certain food groups. Overall, these results may have relevant implications for clinical practice, as they support the integration of specific nutritional recommendations in the clinical follow-up of people with Down syndrome and guide caregivers and/or family members in promoting healthier lifestyles.

## Supplemental Information

10.7717/peerj.20597/supp-1 Supplemental Information 1 Raw data.

10.7717/peerj.20597/supp-2 Supplemental Information 2 Codebook.

10.7717/peerj.20597/supp-3 Supplemental Information 3 Strobe Checklist.

## Additional Information and Declarations

### Data Availability

The following information was supplied regarding data availability:

The data is available in the Supplemental File.

The data is available at Arias Montano: https://hdl.handle.net/10272/25543.
