# Supplementary material for: Influence of posture during mastication on body composition and nutritional intake in individuals with Down syndrome
Source: PeerJ. 2026 Jan 15;14:e20597. doi: 10.7717/peerj.20597 (PMC12812275; doi:10.7717/peerj.20597)
Supplement: Supplemental Information 2 [file peerj-14-20597-s002.docx]

| **Variable name** | **Description** | **Measure** | **Codes** |
| --- | --- | --- | --- |
| ID | Participant identifier number | Nominal |  |
| Sex | Sex |  | 1=Men; 2= Women |
| Age | Age | Years |  |
| Height | Height | Centimeters |  |
| Weight | Weight | Kilograms |  |
| BMI | Body Mass Index | Kg/m² |  |
| BMI-recoded | Body Mass Index recoded | Kg/m² | 1= Insufficient weight  2= Healthy weight  3= Overweight  4= Obesity |
| Basal metabolism | Basal metabolism | Kcal/day |  |
| Fat Mass | Fat Mass | Kilograms |  |
| % Fat Mass | Percentage of fat mass | Percentage |  |
| Fat-free mass | Fat-free mass | Kilograms |  |
| % Fat-free mass | Percentage of fat free mass | Percentage |  |
| Lean mass | Lean mass | Kilograms |  |
| Bone mineral | Bone mineral | Kilograms |  |
| Dwelling | Dwelling |  | 1=Dependent dwelling  2= Independent dwelling |
| Altered mastication posture | Altered mastication posture |  | 1= Present  2=Absent |
| Protides (g) | Protides | Grams |  |
| Glucids (g) | Glucids | Grams |  |
| Lipids (g) | Lipids | Grams |  |
| Total sugar | Total sugar | Grams |  |
| Fiber (g) | Fiber | Grams |  |
| Saturated fats (g) | Saturated fats | Grams |  |
| Cholesterol (g) | Cholesterol | Grams |  |
| Vitamin Aµg | Vitamin A | Micrograms |  |
| B1mg | Vitamin B1 | Milligrams |  |
| B2mg | Vitamin B2 | Milligrams |  |
| B3mg | Vitamin B3 | Milligrams |  |
| B6mg | Vitamin B6 | Milligrams |  |
| B12µg | Vitamin B12 | Micrograms |  |
| Vitamin Cmg | Vitamin C | Milligrams |  |
| Vitamin Dµg | Vitamin D | Micrograms |  |
| Vitamin Emg | Vitamin E | Milligrams |  |
| Vitamin Kµg | Vitamin K | Micrograms |  |
| Namg | Sodium | Milligrams |  |
| Kmg | Potassium | Milligrams |  |
| Camg | Calcium | Milligrams |  |
| Mgmg | Magnesium | Milligrams |  |
| Pmg | Phosphorus | Milligrams |  |
| Cumg | Copper | Milligrams |  |
| Znmg | Zinc | Milligrams |  |
| Mnmg | Manganese | Milligrams |  |
| Packaged juices per day (g) | Packaged juices per day | Grams |  |
| Eggs and egg products per day (g) | Eggs and egg products per day | Grams |  |
| Sausages per day (g) | Sausages per day | Grams |  |
| Vegetables per day (g) | Vegetables per day | Grams |  |
| Legumes per day (g) | Legumes per day | Grams |  |
| Fresh fruit daily (g) | Fresh fruit daily | Grams |  |
| Rice and seeds per day (g) | Rice and seeds per day | Grams |  |
| Pasta per day (g) | Pasta per day | Grams |  |
| Whole milk per day (g) | Whole milk per day | Grams |  |
| Skimmed milk per day (g) | Skimmed milk per day | Grams |  |
| Bread and bread products per day (g) | Bread and bread products per day | Grams |  |
| Cereals and tubers per day (g) | Cereals and tubers per day | Grams |  |
| White meat per day (g) | White meat per day | Grams |  |
| Beef/veal and by-products per day (g) | Beef/veal and by-products per day | Grams |  |
| Pork and pork products per day (g) | Pork and pork products per day | Grams |  |
| Lamb per day (g) | Lamb per day | Grams |  |
| High-calorie foods per day | High-calorie foods per day | Grams |  |
| White fish per day (g) | White fish per day | Grams |  |
| Blue fish per day (g) | Blue fish per day | Grams |  |
| Crustaceans, shellfish or mollusks per day (g) | Crustaceans, shellfish or mollusks per day | Grams |  |
| Sugars and sweets per day (g) | Sugars and sweets per day | Grams |  |
| Chocolate and derivatives per day (g) | Chocolate and derivatives per day | Grams |  |
| Spices and flavorings per day (g) | Spices and flavorings per day | Grams |  |
| Flour per day (g) | Flour per day | Grams |  |
| Oils and fats per day (g) | Oils and fats per day | Grams |  |
