# Supplementary material for: Influence of posture during mastication on body composition and nutritional intake in individuals with Down syndrome
Source: PeerJ. 2026 Jan 15;14:e20597. doi: 10.7717/peerj.20597 (PMC12812275; doi:10.7717/peerj.20597)
Supplement: Supplemental Information 3 [file peerj-14-20597-s003.doc]

STROBE Statement—Checklist of items that should be included in reports of ***cross-sectional studies***

|  | Item No | Recommendation |
| --- | --- | --- |
| **Title and abstract** | 1 | (*a*) Indicate the study’s design with a commonly used term in the title or the abstract. **Page 1. Lines 22.** |
|  |  | (*b*) Provide in the abstract an informative and balanced summary of what was done and what was found. **Page 1. Lines 17 - 30.** |
| Introduction | | |
| Background/rationale | 2 | Explain the scientific background and rationale for the investigation being reported. **Page 1-2. Lines 35-72.** |
| Objectives | 3 | State specific objectives, including any prespecified hypotheses. **Page 2-3 Lines 73-82.** |
| Methods | | |
| Study design | 4 | Present key elements of study design early in the paper. **Page 3. Lines 84-97.** |
| Setting | 5 | Describe the setting, locations, and relevant dates, including periods of recruitment, exposure, follow-up, and data collection. **Page 3. Lines 84-97.** |
| Participants | 6 | (*a*) Give the eligibility criteria, and the sources and methods of selection of participants. **Page 3. Lines 84-97.** |
| Variables | 7 | Clearly define all outcomes, exposures, predictors, potential confounders, and effect modifiers. Give diagnostic criteria, if applicable. **Page 3. Lines 92-94.** |
| Data sources/ measurement | 8* | For each variable of interest, give sources of data and details of methods of assessment (measurement). Describe comparability of assessment methods if there is more than one group. **Page 3-4. Lines 101-157.** |
| Bias | 9 | Describe any efforts to address potential sources of bias. **Page 3. Line 120.**  **Page 4. Lines 130-140.** |
| Study size | 10 | Explain how the study size was arrived at. **Page 3. Line 86.** |
| Quantitative variables | 11 | Explain how quantitative variables were handled in the analyses. If applicable, describe which groupings were chosen and why. **Page 4-5. Lines 159-173.** |
| Statistical methods | 12 | (*a*) Describe all statistical methods, including those used to control for confounding. **Page 4-5. Lines 159-173. Statistical analyses were descriptive and comparative between groups using nonparametric tests (Mann-Whitney U, Kolmogorov-Smirnov and chi-square). No statistical adjustment was made to control for possible confounding variables.** |
|  |  | (*b*) Describe any methods used to examine subgroups and interactions. **Page 4-5. Lines 159-173.** |
|  |  | (*c*) Explain how missing data were addressed. **No missing data** |
|  |  | (*d*) If applicable, describe analytical methods taking account of sampling strategy**. A non-probabilistic convenience sampling method was used; therefore, no specific analytical adjustments based on the sampling strategy were necessary.** |
|  |  | (*e*) Describe any sensitivity analyses. **Not applicable** |
| Results | | |
| Participants | 13* | (a) Report numbers of individuals at each stage of study—eg numbers potentially eligible, examined for eligibility, confirmed eligible, included in the study, completing follow-up, and analysed. **Flow diagram included. Page 3, line 99** |
|  |  | (b) Give reasons for non-participation at each stage. **Not applicable** |
|  |  | (c) Consider use of a flow diagram. **Page 3. Line 99.** |
| Descriptive data | 14* | (a) Give characteristics of study participants (eg demographic, clinical, social) and information on exposures and potential confounders. **Page 5. Lines 176-185.** |
|  |  | (b) Indicate number of participants with missing data for each variable of interest**. There are no missing data for the variables of interest.** |
| Outcome data | 15* | Report numbers of outcome events or summary measures. **Page 5. Lines 189-190.** |
| Main results | 16 | (*a*) Give unadjusted estimates and, if applicable, confounder-adjusted estimates and their precision (eg, 95% confidence interval). Make clear which confounders were adjusted for and why they were included.  **In the results section, we report unadjusted estimates comparing groups with and without postural alteration during mastication, including median differences and p-values from nonparametric tests. Adjusted analyses controlling for potential confounders were not performed due to the exploratory nature of the study and the limited sample size. Therefore, no confounder-adjusted estimates or confidence intervals are presented.** |
|  |  | (*b*) Report category boundaries when continuous variables were categorized. **The adequacy or inadequacy of intake - Page 4. Lines 123-129, it is not included in the results, it is explained in the method. BMI - Page 5. Lines 180-181.** |
|  |  | (*c*) If relevant, consider translating estimates of relative risk into absolute risk for a meaningful time period. **As this is a cross-sectional study without longitudinal follow-up, relative risk estimates and their conversion to absolute risk over time are not applicable** |
| Other analyses | 17 | Report other analyses done—eg analyses of subgroups and interactions, and sensitivity analyses. **No additional analyses, such as subgroup, interaction, or sensitivity analyses, were performed in this study**. |
| Discussion | | |
| Key results | 18 | Summarise key results with reference to study objectives. **Page 7. Lines 248-266.** |
| Limitations | 19 | Discuss limitations of the study, taking into account sources of potential bias or imprecision. Discuss both direction and magnitude of any potential bias. **Page 10. Lines 373-380.** |
| Interpretation | 20 | Give a cautious overall interpretation of results considering objectives, limitations, multiplicity of analyses, results from similar studies, and other relevant evidence. **Page 7-10. Lines** **267-368.** |
| Generalisability | 21 | Discuss the generalisability (external validity) of the study results. **Page 10. Lines 369-372.** |
| Other information | | |
| Funding | 22 | Give the source of funding and the role of the funders for the present study and, if applicable, for the original study on which the present article is based. **Funding appears in the declarations section during manuscript submission.** |

*Give information separately for exposed and unexposed groups.

**Note:** An Explanation and Elaboration article discusses each checklist item and gives methodological background and published examples of transparent reporting. The STROBE checklist is best used in conjunction with this article (freely available on the Web sites of PLoS Medicine at http://www.plosmedicine.org/, Annals of Internal Medicine at http://www.annals.org/, and Epidemiology at http://www.epidem.com/). Information on the STROBE Initiative is available at www.strobe-statement.org.
